# Editorial: Valorization of bioactive compounds from bio-wastes of agro-food sector using green technologies

**DOI:** 10.3389/fnut.2023.1334315

**Published:** 2023-11-24

**Authors:** Najla Trabelsi, Ioannis Mourtzinos, Mohamed Fawzy Ramadan

**Affiliations:** ^1^Center of Biotechnology of BorjCedria, LR15CBBC05 Olive Biotechnology, Hammam-Lif, Tunisia; ^2^Department of Food Science and Technology, Faculty of Agriculture, Aristotle University of Thessaloniki, Thessaloniki, Greece; ^3^Department of Clinical Nutrition, Faculty of Applied Medical Sciences, Umm Al-Qura University, Makkah, Saudi Arabia

**Keywords:** byproducts, waste, phytochemicals, bioactivity, circular economy, technology, industry, environment

The term “waste” refers to items in our immediate environment that should be recycled, reused, decreased, or even removed if feasible. Global waste is predicted to eventually amount to 3.40 billion tons by 2050. Nevertheless, bio-waste may be seen as a source of environmental toxins or pollutants, depending on how it is handled. Large volumes of bio-waste from the agri-food sector are created these days, stressing the environment. Bio-wastes derived from agri-food are frequently produced at every stage of the food supply chain, from harvest to disposal. The phases and the food product determine the different quantities. For instance, European homes account for 53% of bio-waste, with processing accounting for 19% of all food waste. Production (11%) and wholesale and retail (5%), along with catering (12%), account for the remaining 28%. As a result, it has become clear that a shift toward a sustainable circular economy is necessary.

Ensuring the industry's economic, social, and environmental sustainability requires improving waste management. Agro-food by-product revalorization may be influenced by the concept of a “*Zero Waste Economy*,” where waste is converted into valuable goods and finds practical applications by offering potential energy savings. Sustainability concerns related to industrialization and the economy are addressed by agro-food bio-waste management. The European Commission sought to advance the circular economy. Businesses predicted that when the Green Deal was unveiled, which aimed to make Europe carbon neutral by 2050, bio-waste and its harmful environmental effects would decline in the future. The “*Farm to Fork*” plan also aims to integrate circular economy concepts into food processing successfully.

The agricultural food industry's biowastes are abundant in hydrophilic and lipophilic phytochemicals. Numerous phytochemicals, including vitamins, carotenoids, pigments, phenolics, and tocols, may improve the nutritional value of food. Because of their superior nutritional worth, the enriched products may also be designated as functional foods. Phytochemicals recovered from agro-food bio-wastes can potentially extend the shelf life of various products, including food, cosmetics, and pharmaceuticals. The phytochemicals' antioxidant and antibacterial traits may also enhance these benefits. These days, a global strategy aims to produce value-added products for potential uses like food additives or coloring, bio-fertilizers, biodiesel, and nutraceuticals through the sustainable utilization of agro-food bio-wastes. According to this viewpoint, choosing the best extraction method and specifying the intended applications are crucial for improving the value of agro-food bio-wastes. Meanwhile, unique environmental-friendly methods, including ultrasound-assisted extraction (UAE), microwave-assisted extraction (MAE), and supercritical fluid extraction (SFE), have been developed for the sustainable recovery of phenolics from agro-food bio-wastes. In addition, a new generation of sustainable green solvents has been used (1–5).

This Research Topic is aimed at collecting papers suitable to improve our knowledge and understanding of the extraction, characterization, and applications of bioactive compounds from agro-food bio-wastes, considering their biological traits and use in nutraceuticals, food enrichment, preservation or packaging, or for pharmaceutical and cosmetic applications. Potential subtopics included (1) novel techniques for green extraction of bioactive compounds from agro-food waste, (2) characterizing natural compounds from by-products and evaluating biological properties in correlation with food applications and human health benefits, and (3) new systems for the administration of by-product biomolecules, in correlation with the application in food products.

Four articles covering the aspects mentioned above were published in this Research Topic. The first article reviewed the potential of *Psidium guajava* by-products in traditional Mexican medicine (Gutierrez-Montiel et al.). In this review, the authors concluded that the valorization of *Psidium guajava* bio-waste may benefit the agroindustry economically and could represent an alternative to traditional antimicrobials. The second research article reported on deep eutectic solvent-ultrasound assisted extraction (UAE-DES) for enhanced extraction of naringenin from *Searsia tripartita* and retained their bioactivities (El Maaiden et al.) In this research, the UAE-DES technique produced high-efficiency naringenin extraction while retaining bioactivity, implying broad application potential and making it a high-throughput green extraction method worthy of consideration. In the third research article (Mkhari et al.), the authors studied the encapsulation of betalain-rich extract from beetroot bio-waste using a blend of gum Arabic and maltodextrin to promote a food circular economy. They highlighted that although blending gum Arabic and maltodextrin has the potential to improve the quality of beetroot waste extract powder, using these biopolymers separately showed a promise to promote a food circular economy. In the last research article (Elkatry et al.), the authors studied the potential use of Indian rice flour or husk to fortify pan bread. They highlighted the possible use of rice husk fibers in baking goods and the possible health benefits while contributing to the long-term use of agricultural waste. Meanwhile, the results reported in those studies highlighted and reviewed new relevant data on the valorization of bioactive compounds from bio-wastes of the agro-food sector using green technology.

## Author contributions

NT: Writing – original draft. IM: Writing – original draft. MFR: Writing – original draft, Writing – review & editing.
